# Quality of Life in Patients With Tongue Cancer After Surgical Treatment: A 12-Month Prospective Study

**DOI:** 10.7759/cureus.22511

**Published:** 2022-02-23

**Authors:** Dimitra Palitzika, Ioannis Tilaveridis, Maria Lavdaniti, Konstantinos Vahtsevanos, Angeliki Kosintzi, Konstantinos Antoniades

**Affiliations:** 1 Nursing, Papanikolaou Hospital, Thessaloniki, GRC; 2 Oral and Maxillofacial Surgery, Aristotle University of Thessaloniki, Thessaloniki, GRC; 3 Nursing, International Hellenic University, Thessaloniki, GRC; 4 Oral and Maxillofacial Surgery, Papanikolaou Hospital, Aristotle University of Thessaloniki, Thessaloniki, GRC; 5 Special Education, Aristotle University of Thessaloniki, Thessaloniki, GRC

**Keywords:** cancer stage, local, flap, tumor, surgical excision, function, quality of life, cancer, tongue, oral

## Abstract

Aim of the study

The project assessed the quality of life in post-operative patients with tongue cancer at three-month intervals in the first year after the operation.

Material and methods

A longitudinal prospective cohort study was conducted in the oral and maxillofacial department of a large public general hospital in northern Greece. Fifty-six patients out of a total of 156 with oral cancer were chosen for the study, who completed distinct quality-of-life surveys (EORTC QLQ-C30, and QLQ Head & Neck Module FACT-G).

Results

Tumor size correlated negatively with various EORTC QLQ-C30 scores, such as role functioning (p = 0.004) and cognitive functioning (p = 0.007), in the third evaluation. Tumor size correlated positively with subscale problems such as social eating (p = 0.001) and weight loss (p = 0.004) in the QLQ Head & Neck Module. The role functioning subscale (p = 0.003), the pain subscale (p = 0.001), and the speech issues QLQ Head & Neck module subscale (p = 0.003) adversely correlated with cancer stage. Patients who received flap reconstruction significantly differed from those who did not, on the EORTC QLQ-C30 cognitive functioning (U = 139.0, p = 0.006), dyspnea (U = 391.5, p = 0.006), and diarrhea (U = 425.0, p = 0.007) subscales during the third evaluation. Differences were also found in the QLQ-H&N35 subscale of sticky saliva (U = 391.0, p = 0.006).

Patients with flap reconstruction did not significantly differ from those with immediate closure after one year. Differences concerned the EORTC QLQ-C30 subscales of cognitive functioning, dyspnea, and diarrhea, and the QLQ Head & Neck Module subscale of sticky saliva on the third assessment. No statistically significant correlations were observed between tumor size and cancer stage in the fourth assessment, but the grade of cancer positively correlated with the EORTC QLQ-C30 subscale of constipation (p = 0.000).

Conclusions

Our study suggests that quality of life is impaired in patients with tongue cancer who have undergone surgical interventions, particularly within the first month post-operation. However, quality of life is fully restored one year after the surgical excision. Future studies should explore early interventions to help healthcare providers better treat this unique group of patients.

## Introduction

Oral cavity cancer is the most common malignancy in the head and neck region, according to the Global Cancer Observatory (GLOBOCAN). By 2030, the global incidence of new oral cavity cancer is predicted to approach 29/100,000 people in men and women of all ages [1]. The most prevalent oral cavity cancer form is the squamous cell carcinoma of the tongue, whose prevalence has grown in the last three decades, to 3.0 per 100,000 people but remains more common in senior men than in women or younger people [2]. Tongue cancer’s aggression can be attributed to its silent quick progression from premalignant to invasive carcinoma. Diagnosis is often delayed, resulting in a bad prognosis [3].

Surgery is the preferred treatment for squamous cell carcinoma of the tongue. A histopathological margin distance of less than 5 mm (Free Margin Status) is crucial for local control and disease-free survival [4]. Patients who have positive resection margins or close margins with poor growth characteristics are given adjuvant treatment, which can include wider resection in a second operation or chemoradiotherapy [5]. Disease-free survival and overall long-term survival are the primary metrics of how successful cancer treatment is, though response rates to treatment, relapse or treatment failure, mortality, and length of hospital stay are also factors. Psychological factors, such as quality of life (QOL), are also crucial [6].

QOL describes a patient’s overall well-being and is, by definition, multidimensional, as it includes physical, social, and emotional functioning domains from the patient’s point of view [7,8]. Unsurprisingly, surgical treatment of tongue cancer influences a patient’s QOL [9], including eating, speaking, breathing, and physical appearance.

Improving QOL should be recognized as a final treatment goal [10], because head and neck cancer causes more somatic and psychological discomfort than other cancers; these cancers impair self-image, self-esteem, confidence, and identity to a higher extent than less obvious malignancies. Untreated distress can have long-term negative effects on patients’ desire to survive and improve their QOL [11]. As a result, the focus of our research is on quality of life.

Although several studies have characterized the QOL of head and neck cancer patients, such reports on those with tongue cancer are scarce [7]. No study has examined QOL in this group of patients after surgical treatment in Greece to date.

A patient’s QOL one year after cancer treatment is a good long-term predictor of QOL in disease-free individuals [12]. Thus, this study assessed tongue cancer patients’ QOF before surgery, as well as three months, six months, and one year after surgery, and explored which factors influence their QOL.

## Materials and methods

Study design and sample

The longitudinal study was conducted in the Department of Oral & Maxillofacial Surgery, of a public hospital in Thessaloniki, Greece. Data were collected between October 2016 and November 2019 from 56 patients, who were selected from a total of 135 head and neck cancer patients. All patients gave written informed consent before completing questionnaires, and their participation was completely voluntary. Formal permission was obtained from the administration and the hospital’s ethics committee to conduct the research project and use the clinic’s archives to acquire additional patient information. Inclusion criteria included age over 18 years, cancer diagnosis, no previous neurological and/or psychiatric history, ability to comprehend and write in Greek, and current mental competence to communicate sufficiently.

Patients completed the surveys four times. Prior to surgery, a baseline assessment was performed. Patients completed surveys four times: baseline as well as three, six, and 12 months following surgery. Fifty-six out of a total of 135 participants attended all four sessions.

Instruments

A sociodemographic and clinical questionnaire designed for this study includes two separate QOL questionnaires as well as one functional impairment scale.

The Greek version of the European Organization for Research and Treatment of Cancer Quality of Life Questionnaire, version 3.0, was used (EORTC QLQ-C30) and included five functional scales, three symptoms scales, a global health status/quality of life scale, and six single items, whose scores all ranged from 0-100 [13]. Higher functional scale scores are connected with higher health-related quality of life (HRQOL), but higher symptom scale/item scores implied a higher level of symptoms [14].

The EORTC QLQ-C30 can be combined with supplementary questionnaire modules to provide more details on specific clinical populations. We used the EORTC QLQ-H&N35, a 35-item questionnaire, for the current study’s head and neck-specific module [15]. Seven multiple-item symptom scales (pain, swallowing, taste/smell, speech, social eating, social contacts, and sexuality) and six symptom items (teeth problems, trismus, dry mouth, sticky saliva, cough, and feeling ill) were included [16]. The EORTC QLQ-C30 and QLQ-H&N35 have both been tested and validated for the Greek population and deemed sufficiently valid and reliable [13,15].

The second measure of QOL was the Functional Assessment of Cancer Therapy Scale - General (FACT-G), which consists of 27 items grouped into four domains of QOL (physical, social, emotional, and functional). The FACT-G also calculates a total QOL score by adding the four subscale ratings. This questionnaire was validated in the past for the Greek population [17].

Finally, the Karnofsky Performance Status Scale was used to assess functional impairment with a single score from 0-100 [18]. It has been used many times in medical research involving the Greek population [19,20].

Data analysis

Statistical analyses were performed using IBM SPSS Statistics 25 (IBM Corp., Armonk, NY). The Kolmogorov-Smirnov test was performed to determine distribution normality. Skewness and kurtosis were also considered. Non-parametric tests were chosen because the data distribution did not match the criteria for normality. Gender differences were investigated using the Mann-Whitney test. Spearman’s correlation and Kruskal-Wallis test were used to measure correlations and investigate demographic disparities, respectively. After testing for homogeneity with Leven’s test, linear regression was used to characterize the effects of clinical variables on performance status.

## Results

Cronbach's alpha ranged from 0.72 to 0.87 for the EORTC QLQ-C30, QLQ-H&N35, and FACT-G scales and subscales in this study.

The demographic characteristics of 56 patients included in the study are shown in Table 1.

**Table 1:** Patient demographics.

Variables	n	%
Gender		
Male	31	55.4
Female	25	44.6
Family status		
Single	4	7.4
Married/With partner	27	50.0
Divorced	5	9.3
Widowed	18	33.3
Education		
No education (illiterate)	1	1.9
Primary school (6 years)	27	50.0
Middle school (9 years)	8	14.8
High school (12 years)	10	18.5
University (16 years)	7	13
Master’s/PhD	1	1.9
Occupation		
Unemployed	7	13
Private sector	7	13
Public sector	3	5.6
Self-employed	5	9.3
In retirement	32	59.3
Residence		
City	31	57.4
Town	5	9.3
Village	18	33.3

The mean age of the population was 65.2 (± 13.2) years, with a range from 32 to 91 years of age. Most participants were male (55.4%), and many were married or with a partner (48.2%). Most participants were in retirement (57.1%) and lived in a city (57.4%).

Clinical features indicated that over half of the patients (53.6%) underwent surgery. Most had lymphadenectomy (98.3%); 77.804% had selective neck dissection, and 17.9% had modified radical neck dissection, levels I-V (MRND I-V). Most patients were in the fourth stage (43.8%), though 88.5% were in grade 1 or 2 (Table 2).

**Table 2:** Clinical characteristics of the sample. MRND I-V: modified radical neck dissection, levels I-V

Variables	n	%
Lymphadenectomy		
No	1	1.8
Selective neck dissection	45	80.4
MRND I-V	10	17.9
Stage		
I	5	10.4
II	17	35.4
III	5	10.4
IV	21	43.8
Grade		
1	27	51.9
2	19	36.5
3	6	11.5
Size		
1	6	13.3
2	20	44.4
3	4	8.9
4	15	33.3
Flap		
No	41	73.2
Yes	15	26.8

The yearly assessment revealed that 93.5% of the patients scored over 90 on the Karnofsky Performance Status Scale, and 96.8% scored over 80. A total of 71% of participants scored over 80 and 77.4% over 75, on the global health scale. A total of 80% of participants scored over 90 on the physical functioning scale, and 61.3% scored over 80 on the emotional functioning scale. On the role functioning scale, 61.3% of participants scored over 80. The cognitive functioning and social functioning scales showed similar levels, with 93.5% and 71% of participants, respectively, scoring over 80. After one year, the majority of participants reported reduced symptoms, scoring below 30 on the head and neck module (QLQ-H&N35). On the pain and feeling ill scales, 83.9% and 93.5% of patients scored below 16.7 and 33.3, respectively.

Tumor size negatively correlated with some of the EORTC QLQ-C30 scales, such as role functioning (rho = -0.538, p = 0.004) and cognitive functioning (rho = - 0.510, p = 0.007), on the third assessment. Tumor size also positively correlated with the subscale problems of social eating (rho = 0.623, p = 0.001) and weight loss (rho = 0.557, p = 0.004) on the QLQ-H&N35. Cancer stage negatively correlated with the role subscale (rho = -0.524, p = 0.003), but positively correlated with the pain subscale (rho = - 0.555, p = 0.001), QLQ-H&N35 speech problems (rho = 0.526, p = 0.003), and trouble with social contact subscales (rho = 0.478, p = 0.008). However, the fourth assessment found no statistically significant relation between tumor size and stage, though a positive correlation was found between cancer grade and the EORTC QLQ-C30 subscale of constipation (rho = 0.643, p = 0.000). Statistically significant results according to Spearman's correlations between subscales and clinical variables on the third assessment are shown in Table 3.

**Table 3:** Results of third assessment.

	Size		Stage			Grade		
EORTC QLQ-C30	rho	p		rho	p		rho	p
Role functioning	-0.538	0.004		-0.524	0.003		-0.079	0.657
Cognitive functioning	-0.510	0.007		-0.445	0.014		-0.002	0.992
Pain	0.379	0.051		0.555	0.001		0.188	0.288
Constipation (4^th^ assessment)	-0.022	0.922		0.136	0.507		0.643	0.000
EORTC QLQ-H&N35								
Speech problems	0.450	0.019		0.526	0.003		-0.130	0.464
Trouble with social eating	0.623	.0001		0.445	0.014		-0.010	0.954
Trouble with social contact	0.287	0.146		0.478	0.008		0.047	0.793
Weight loss	0.557	0.004		0.434	0.017		0.000	1.000

We also evaluated the differences between patients who had a surgical flap conventional or free flap and those who did not receive a surgical flap. The third evaluation showed statistically significant differences between the groups with respect to the EORTC QLQ-C30 cognitive functioning (U = 139.0, p = 0.006), dyspnea (U = 391.5, p = 0.006), and diarrhea (U = 425.0, p = 0.007) subscales, as well as the QLQ-H&N35 subscale of sticky saliva (U = 391.0, p = 0.006). The flap reconstruction group reported lower cognitive functioning and more symptoms of dyspnea, diarrhea, and sticky saliva six months after the surgery. The use of a surgical flap did not yield statistically significant results on the fourth assessment. Statistically significant results between flap and non-flap patient groups on the third assessment are shown in Table 4.

**Table 4:** Results according to the type of reconstruction.

	With flap	Without flap		
EORTC QLQ-C30	Mean (SD)	Mean (SD)	U	p
Cognitive functioning	84.8 (13.9)	96.0 (10.0)	139.0	0.006
Dyspnoea	33.3 (21.1)	13.3 (25.5)	391.5	0.006
Diarrhoea	9.1 (15.6)	0.0 (0.0)	425.0	0.007
EORTC QLQ-H&N35				
Sticky saliva	54.5 (27.0)	30.7 (19.1)	391.0	0.006

## Discussion

Tongue cancer treatments have advanced in recent years, yet these therapies continue to damage patients’ cosmetic, physical, psychological, and social functioning [7]. This longitudinal study surveyed tongue cancer patients to better understand which factors impair their QOL post-surgery.

The majority of patients reached a good level of QOL after one year post-surgery. Tumor size and grade significantly correlated with various subscales on the third assessment but with only one subscale (constipation) on the fourth (one-year follow-up). These findings agree with other studies where functionality and QOL are largely restored 12 months after surgery [7,21]. Yang et al. found that overall QOL improved significantly one year following surgery but did not reach pre-operation levels [7]. Borggreven et al. also found that health-related quality of life (HRQOL) concerns reverted to pre-treatment levels at twelve in a well-defined sample of head and neck cancer patients who underwent reconstructive surgery for advanced oral or oropharyngeal cancer [22]. Indeed, surgical treatment for oral or oropharyngeal cancer, especially when combined with radiotherapy, reduces physical functioning and symptoms throughout the first year. Many patients still feel significantly worse than before despite symptoms clearing after one year post-surgery [22,23].

Here, most participants reported reduced symptoms after one year. Gender, age, and other demographic variables did not affect QOL at any stage. This finding contradicts a previous study in which men and older patients with oral cancer scored worse. In this study, patients with a greater degree of education also rated various aspects of their own health status more severely, though there was no correlation between the level of education and any aspect of QOL. However, the patient’s clinical status predicted self-reported QOL better than socio-demographic characteristics [24].

The present study also found that clinical factors - tumor size, grade, and the use of flap reconstruction - affected QOL domains and symptoms. Similarly, Zuydam et al. found that clinical characteristics such as tumor size, site, staging, radiation, type of surgery, and degree of resection of posterior tongue and soft palate determined speech and swallowing scores [25].

Staging criteria, which include the size of the tumor and the extent of metastatic dissemination of the primary lesion, help clinicians to identify treatment options and prognosis for oral cancer [26]. A surgical technique must first be selected, where good surgical margins and long-term survival are primary goals. The location and extent of the invasion, the depth of infiltration, and proximity to the mandible or maxilla influence surgical options. Oral cavity issues such as trismus, dentition, tongue mobility, and the size of the oral aperture, as well as other characteristics such as dentition, size of the oral aperture, degree of mouth opening, and the size and mobility of the tongue, should be considered [27]. Radical surgical therapy often diminishes oral functions and subsequently speech, swallowing, chewing, oral rehabilitation, nutrition, and appearance being issues of particular importance [28]. Surgical treatment-related scarification and altered facial structures, as well as functional degeneration of intraoral tissues, may lead to social and economic difficulties [28]. These could explain our results, where tumor size negatively correlated with various EORTC QLQ-C30 scales, such as role functioning and cognitive functioning on the third evaluation. The QLQ-H&N35 subscales of social eating and weight loss were also significantly linked to tumor size. Cancer stage negatively correlated with the role subscale but positively correlated with the pain subscale, as well as with the QLQ-H&N35 speech problems and trouble with social contact subscales. We found no statistically significant correlations between tumor size and stage on the fourth assessment, although there was a positive correlation between cancer grade and the EORTC QLQ-C30 subscale of constipation.

No significant differences were found at six months post-surgery, between patients who received flap reconstruction and those who had not. This finding is consistent with a recent study that compared the QOL across groups of patients with advanced oral cavity tumors after mandibular resection: those who underwent plate reconstruction, those who underwent flap reconstruction, and those who did not receive any reconstruction. Patients with flap reconstruction showed improved function and fewer problems [29]. On the other hand, others showed that primary closure effected equivalent or better function than flap repair in patients who underwent oral cavity and oropharyngeal reconstruction [30].

Although this study was conducted in a single hospital and cannot be applied to the Greek population at large, its findings highlight the need for more longitudinal studies on QOL in patients with tongue cancer after surgery. Our work is the first population-based study to evaluate QOL in patients with oral cancer in Greece.

## Conclusions

This study filled a void of QOL assessments in Greek tongue cancer patients. We found that QOL plummets, and patients recorded clinical symptoms in the first few months after surgery. QOL restored to pre-surgical levels after one year. Further research is needed to fully understand this patient group’s treatment needs and challenges. Our findings will help healthcare practitioners develop healthcare programs to improve patients’ QOL.
